# Case Report: Delayed appearance of the ablation margin after CT-guided microwave ablation of a solid pulmonary nodule

**DOI:** 10.3389/fmed.2026.1972087

**Published:** 2026-09-08

**Authors:** Hao Zhang, Yan-Hao Zhu, Yi-En Hong, Dong Lan

**Affiliations:** Department of Radiology, Dianjiang People’s Hospital of Chongqing, Chongqing, China

**Keywords:** computed tomography guidance, ground-glass halo, interventional radiology, microwave ablation, pulmonary nodule

## Abstract

During CT-guided thermal ablation of lung tumors, guidelines define the intraprocedural endpoint as a circumferential margin of increased attenuation extending 5–10 mm beyond the tumor. This criterion does not require the strict radiologic texture of ground-glass opacity. The timely appearance of this margin is the main real-time marker of an adequate treatment volume, and its absence is conventionally read as underablation, prompting additional energy delivery. We report a metastatic solid nodule in the left lower lobe treated with microwave ablation (45 W, 8 min total) via a single antenna, without repositioning. No such margin formed after three ablation cycles or after needle withdrawal, on a scan obtained 21 min after the first cycle and beyond the recommended 5- to 10-minute window; the only intraprocedural change was slight, non-circumferential enlargement of the solid-attenuation area. A well-defined margin with dense consolidation appeared only at 30.5 h; by 32 days, most consolidation had resorbed, forming a cavity. Despite adequate energy delivery, the guideline-defined margin in this solid nodule remained unconfirmed well beyond the standard observation window, and its absence should not be treated as an automatic indication for further ablation.

## Introduction

CT-guided percutaneous thermal ablation is an established local therapy for patients who cannot tolerate or decline surgery. Low-dose CT screening is detecting more small, early-stage malignant nodules amenable to local treatment. For these lesions, ablation achieves oncologic outcomes comparable to thoracoscopic sublobar resection (1). Consensus guidelines require the ablation zone to extend 5–10 mm beyond the tumor as a circumferential margin of increased attenuation, an area conventionally termed ground-glass opacity (GGO) in this literature (2–5). This operational usage denotes any perilesional increase in attenuation forming a measurable margin and does not require the strict radiologic texture the term carries elsewhere, a hazy opacity through which the underlying bronchovascular markings remain visible (6). Regardless of its attenuation pattern, the timely appearance of this margin is the main real-time marker used to judge an adequate treatment volume, reflecting thermal injury such as congestion, exudation, hemorrhage, and coagulative necrosis (7, 8). Its absence is conventionally read as underablation, prompting operators to add further energy.

While guidelines define this margin as the intraprocedural endpoint, they offer no guidance on how to proceed when it fails to form within the standard observation window despite adequate energy delivery. Using this operational definition, margin absence has been reported in roughly 6.6% of subsolid nodules (9). In solid nodules, comparable data are lacking, and the true frequency and management of this scenario remain unclear. We report a case in which the guideline-defined margin did not form during microwave ablation of a metastatic solid nodule: not after three ablation cycles, not after needle withdrawal, and not until approximately 30.5 h later.

## Case report

**Figure 1:**
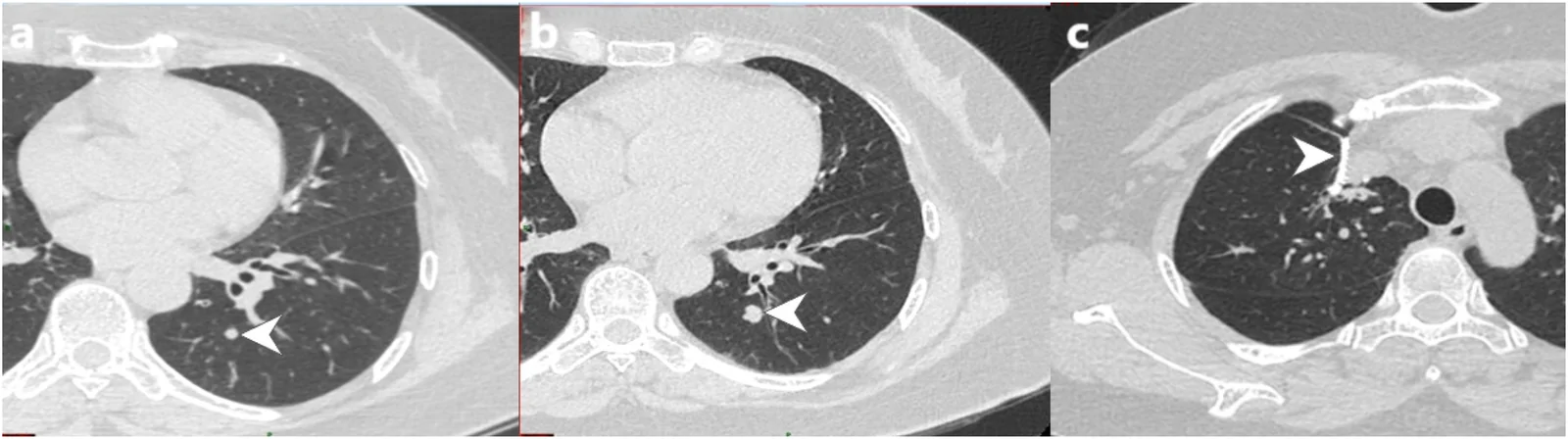
Pre-ablation axial thin-section chest CT (lung window). **(a)** Baseline scan obtained 6 months earlier shows a small, round solid nodule in the dorsal segment of the left lower lobe, with well-defined margins (arrowhead, 6.2 × 5.8 mm). **(b,c)** Follow-up 1 week before ablation: **(b)** the nodule (arrowhead, 10.4 × 7.2 mm) has clearly enlarged compared with baseline; **(c)** at the level of the apical segment of the right upper lobe, a linear high-attenuation staple line from the prior thoracoscopic segmentectomy is seen, with no evidence of local tumor recurrence at the surgical site.

A 59-year-old woman underwent thoracoscopic apical segmentectomy for lung cancer in 2023, with subsequent CT surveillance. In 2025, two thin-section CT scans obtained 6 months apart showed interval enlargement of a nodule in the dorsal segment of the left lower lobe (Figure 1). Given her surgical history, the nodule's progressive growth, and a negative systemic work-up, this was clinically diagnosed as metachronous metastatic disease; the patient declined biopsy. She had no smoking history and had taken no antiplatelet or anticoagulant medication before ablation.

**Figure 2:**
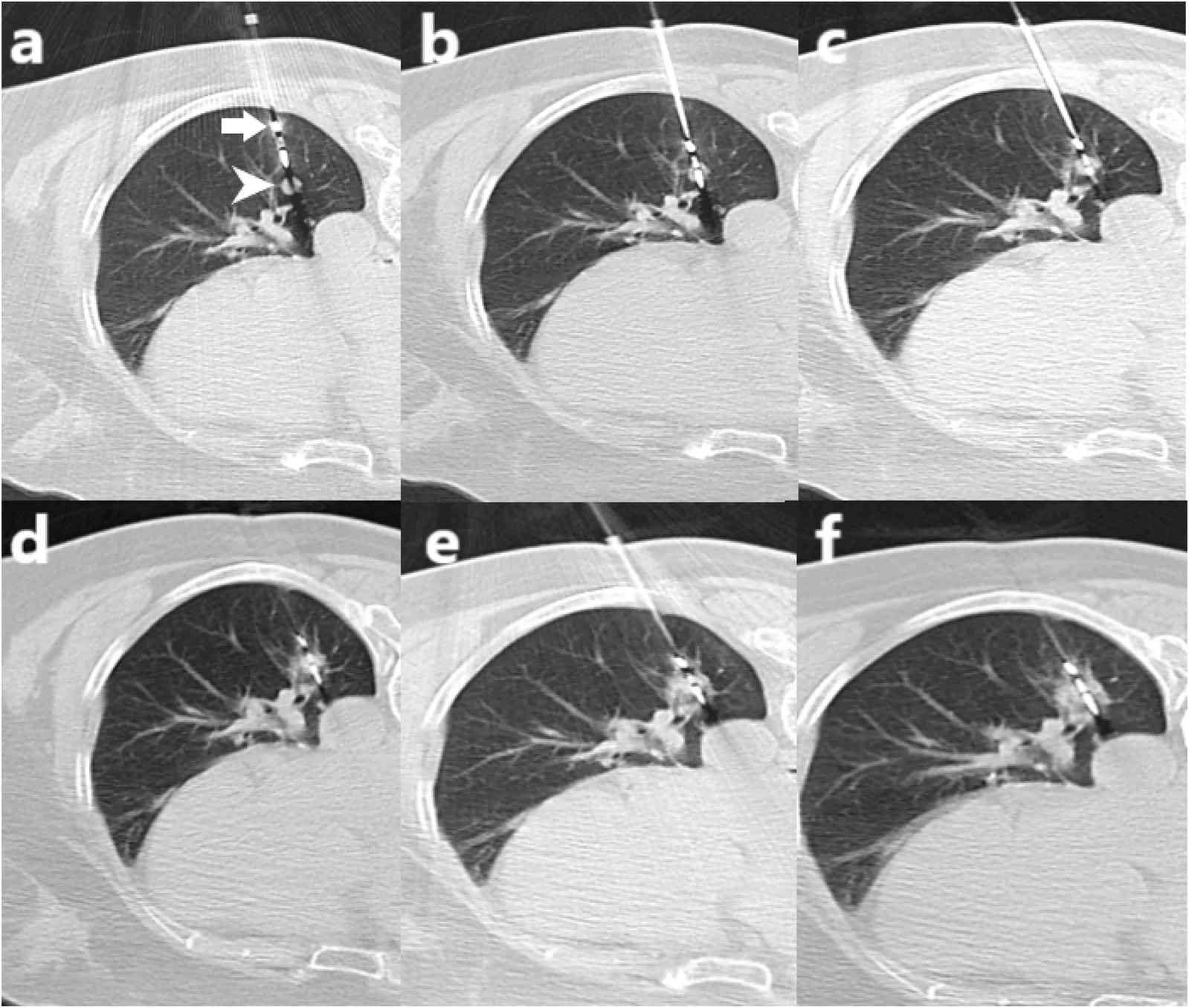
Same-level intraprocedural axial chest CT (lung window). **(a,b)** The needle (arrow) advances along the same track to the target (arrowhead). **(c–e)** Scans after each of three ablation cycles show no circumferential ablation margin in the target zone. **(f)** Delayed observation scan before withdrawal.

Under local anesthesia and prone positioning, a single-use microwave antenna (2450 MHz, 2-mm diameter) was placed via a posterior approach and connected to the matching generator; the needle track and tip position were not adjusted at any point during the procedure. Three ablation cycles were performed at 45 W (3, 3, and 2 min; 8 min total). A rescan was obtained after each cycle. No circumferential margin of increased attenuation appeared after any cycle; the only detectable change was slight, non-circumferential enlargement of the solid-attenuation area. An *ex vivo* heating test confirmed normal antenna output. No further energy was delivered, given concern for thermal injury to the adjacent airway and vessels. The post-withdrawal scan was obtained 21 min after the first cycle ended, beyond the recommended 5- to 10-minute waiting window. It showed no margin and only a trace subpleural pneumothorax that required no treatment. The patient had no chest pain, dyspnea, or hemoptysis during the procedure (Table 1; Figure 2).

**Table 1:** Complete procedural and imaging timeline.

Time from ablation	Event/Key imaging finding
−25 months	Thoracoscopic apical segmentectomy of the right upper lobe for lung cancer
−6 months	Pre-ablation thin-section CT (baseline): small nodule in the left lower lobe (Figure 1a)
−1 week	Pre-ablation thin-section CT: nodule clearly enlarged from baseline (Figure 1b); no recurrence at the prior surgical site (Figure 1c)
0	Needle crosses the pleura; tip 0.5 cm from the nodule (Figure 2a)
+1 min	Tip reaches the target (Figure 2b); Cycle 1 begins: 45 W × 3 min
+8 min	Rescan: no margin (Figure 2c); Cycle 2 begins: 45 W × 3 min, needle position unchanged
+13 min	Rescan: still no margin; solid-attenuation area slightly larger (Figure 2d); *ex vivo* heating test normal; Cycle 3 begins: 45 W × 2 min
+20 min	Rescan: still no margin; solid-attenuation area slightly larger than before (Figure 2e)
+24 min	Delayed observation scan: still no margin (Figure 2f)
+27 min	Post-withdrawal scan: still no margin; trace subpleural pneumothorax, SIR grade 1 (Figure 3a)
+30.5 h	A well-defined margin appears, with dense consolidation in the target zone; pneumothorax resolved (Figure 3b)
+32 days	Most consolidation resorbed; central cavity formed (Figures 3c,d)

Time from ablation is calculated from the moment the needle crosses the pleura. SIR, Society of Interventional Radiology (complication grading standard).

**Figure 3:**
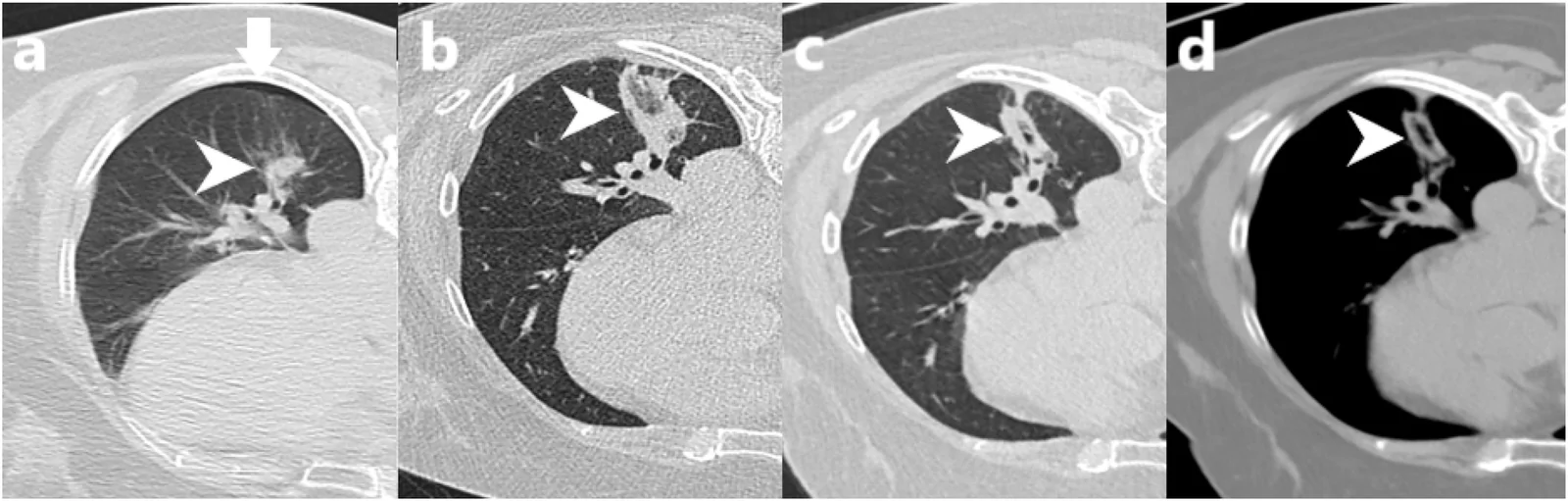
Follow-up axial chest CT **(a–c)**, lung window; **(d)**, mediastinal window). **(a)** Post-withdrawal scan showing a trace of subpleural air (arrow); the target still shows no ablation margin (arrowhead). **(b)** At approximately 30.5 h, a well-defined margin appears around the target zone, with ground-glass change and dense consolidation (arrowhead). **(c,d)** At 32 days, most of the consolidation has resorbed, forming a central cavity (arrowhead), with no pleural effusion or bronchopleural fistula.

The patient received low-flow oxygen and prophylactic antibiotics; her temperature remained normal, and no post-ablation syndrome developed. Follow-up CT at approximately 30.5 h showed a delayed, well-defined margin around the target zone, with extensive ground-glass change and dense consolidation; the pneumothorax had resolved. By 32 days, most of the consolidation had resorbed, forming a cavity with central liquefactive necrosis, without pleural effusion, delayed pneumothorax, or bronchopleural fistula (Figure 3).

## Discussion

As noted in the Introduction, GGO in ablation guidelines is used operationally rather than in its strict radiologic sense (6), a convention the CIRSE standards make explicit by defining GGO as any hyperattenuated opacity corresponding to the tumor and surrounding coagulated lung, regardless of its texture (2). Consensus terminology for the ablation zone itself goes further, making no reference to any specific attenuation pattern and permitting the inner zone to appear solid, honeycomb-like, or bubble-like (10, 11). In this case, the solid-attenuation area of the nodule enlarged slightly during ablation; what remained absent, for an unusually long interval, was a circumferential margin meeting the quantifiable threshold these guidelines specify. Animal studies confirm that tissue within a confirmed margin has lost viability by the time it appears, supporting its use as a surrogate for the necrotic margin (8). Even where present, however, it remains an imperfect marker. It is a composite finding, with a substantial component reflecting intra-alveolar hemorrhage that is difficult to distinguish from ablated tissue on CT because both share similar attenuation (12). It is also time-dependent, becoming visible only once exudation is complete (7), tends to overestimate the true necrotic margin (13), and is inconstant (9).

This pattern has not previously been reported: a solid nodule in which the guideline-defined margin failed to form for such an extended interval. We excluded the usual confounders: premature scanning, equipment malfunction, needle-track displacement, and significant puncture-related hemorrhage. Reported data indicate that 65 W for a median of 2 min produces an immediate ablation zone averaging 3.1 × 2.0 cm (14). Our case delivered a higher cumulative dose (45 W over 8 min), yet showed no margin after three cycles or after withdrawal. A well-defined margin did not appear until 30.5 h. The consolidation pattern was most consistent with thermal coagulation and an acute sterile inflammatory response. Hemorrhage, organizing pneumonia, and infection were less likely, given the absence of fever or hemoptysis and resorption with cavity formation by 32 days (15).

Several factors likely combined to delay the margin's appearance. Aerated lung surrounding the target reduces the contrast between ablation-related vapor and background gas, obscuring the gas bubble typically seen in solid organs (12). It also lowers tissue conductivity and therefore microwave absorption, constraining the margin's visible extent (16). The single, unadjusted needle track, without antiplatelet or anticoagulant therapy, likely minimized the hemorrhagic component that contributes to the margin's formation. Finally, ground-glass nodules show post-ablation change across both tumor and periphery (12). In a solid nodule, the dense, airless center leaves only a thin peripheral rim capable of showing a defined margin.

This margin is a recognized but imperfect surrogate, and current guidelines lack formal guidance for the scenario in which it fails to form within the expected window. We therefore suggest treating its absence as an independent decision point rather than an automatic cue for more energy. First, wait 5–10 min before rescanning (7). Second, check the generator's power log; an *ex vivo* heating test confirms only that the antenna functions normally, not that adequate energy reached the aerated lung. Third, if margin adequacy remains uncertain, reassess the needle-tip-to-tumor geometry and adjust the track if unfavorable, rather than adding energy. Finally, if the margin still cannot be confirmed, we favor terminating the procedure, obtaining follow-up CT at 24–48 h, and maintaining a low threshold for supplemental ablation.

This report is limited by its single-case design, reliance on imaging rather than histologic confirmation of necrosis, follow-up restricted to 32 days on unenhanced CT rather than the 6–12 months needed to confirm technical efficacy (1, 15), and the absence of generator power data.

## Patient Perspective

The patient thanked the operator for exercising caution during the procedure. Faced with an uncertain finding, the operator chose to stop rather than add energy, averting a possible serious complication. She also noted that a malignant pulmonary nodule could now be treated in a single minimally invasive procedure, sparing her a second surgical resection.

## Conclusion

In CT-guided microwave ablation of a solid pulmonary nodule, the guideline-defined ablation margin may remain unconfirmed even after adequate waiting. Recognizing this possibility can avoid unnecessary extension of thermal injury and its associated complications when no reliable imaging endpoint is available.

## Data Availability

The original contributions presented in the study are included in the article/Supplementary Material, further inquiries can be directed to the corresponding authors.
